# Scaffold-mediated switching of lymphoma metabolism in culture

**DOI:** 10.1186/s40170-022-00291-y

**Published:** 2022-10-12

**Authors:** Rachana Bhatt, Dashnamoorthy Ravi, Andrew M. Evens, Biju Parekkadan

**Affiliations:** 1 Department of Biomedical Engineering, Rutgers, The State University of New Jersey, New Brunswick, NJ USA; 2 Division of Blood Disorders, Rutgers Cancer Institute of New Jersey, New Brunswick, NJ USA; 3 Department of Medicine, Robert Wood Johnson Medical School, Rutgers University, New Brunswick, NJ USA; 4 Department of Medicine, Rutgers Biomedical Health Sciences, The State University of New Jersey, New Brunswick, NJ USA

**Keywords:** DLBCL, 3D model, Lymphoma, Proliferation, Metabolic flux, Drug sensitivity

## Abstract

**Background:**

Diffuse large B cell lymphoma (DLBCL) is an aggressive subtype of non-Hodgkin lymphoma (NHL) and accounts for about a third of all NHL cases. A significant proportion (~40%) of treated DLBCL patients develop refractory or relapsed disease due to drug resistance which can be attributed to metabolomic and genetic variations amongst diverse DLBCL subtypes. An assay platform that reproduces metabolic patterns of DLBCL in vivo could serve as a useful model for DLBCL.

**Methods:**

This report investigated metabolic functions in 2D and 3D cell cultures using parental and drug-resistant DLBCL cell lines as compared to patient biopsy tissue.

**Results:**

A 3D culture model controlled the proliferation of parental and drug-resistant DLBCL cell lines, SUDHL-10, SUDHL-10 RR (rituximab resistant), and SUDHL-10 OR (obinutuzumab resistant), as well as retained differential sensitivity to CHOP. The results from metabolic profiling and isotope tracer studies with d-glucose-^13^C_6_ indicated metabolic switching in 3D culture when compared with a 2D environment. Analysis of DLBCL patient tumor tissue revealed that the metabolic changes in 3D grown cells were shifted towards that of clinical specimens.

**Conclusion:**

3D culture restrained DLBCL cell line growth and modulated metabolic pathways that trend towards the biological characteristics of patient tumors. Counter-intuitively, this research thereby contends that 3D matrices can be a tool to control tumor function towards a slower growing and metabolically dormant state that better reflects in vivo tumor physiology.

**Supplementary Information:**

The online version contains supplementary material available at 10.1186/s40170-022-00291-y.

## Background

Metabolic heterogeneity has gained importance as a prognostic biomarker in cancer. Ongoing investigations confirm cross-talk between cancer cell cycle and metabolism [1], conferring additional advantage of escape from host immune surveillance [2]. It is further known that altered metabolism can lead to genomic, transcriptomic, and proteomic variations that affect the tumor microenvironment, malignant transformation, tumor progression, and drug resistance [3, 4].

Metabolic reprogramming is well studied in B-cell lymphoma [5–7] including diffuse large B-cell lymphoma (DLBCL) [8–11]. A recent analysis of fresh tumor specimens by the multi-omics approach has allowed refining DLBCL patient classifications into distinct subtypes and predicting therapies [12]. Based on these studies, what emerges is large variations in intra-patient and intra-tumor metabolic heterogeneity in DLBCL [13, 14], suggesting that metabolic alterations may be a promising target for anti-cancer therapeutics. Indeed, several novel therapeutic strategies focusing on such differential metabolic dependencies have been evaluated in preclinical and early clinical studies [10, 15–17]. However, a salvage chemotherapy regimen still needs to be determined, especially for relapsed DLBCL patients. Thus, it is critical to identify drug leads that affect key driver pathways and combine them rationally to optimize their benefit within a specific patient population.

Conventionally, in vitro studies to understand the basic metabolic reprogramming of DLBCL cell lines are conducted in a two-dimensional (2D) culture [9, 11, 18–20]. On the other hand, it has been observed that cancer cells proliferate at an unnatural pace in 2D and sometimes yield confounding results from experimental investigations [21–23]. The growth kinetics of tumors in a 2D culture and in vivo can differ significantly, in some cases by orders of magnitude. For example, the observed volume doubling time of in vivo tumors was on the order of 100 days or more, in contrast to calculated doubling times of approximately 5 days in 2D experimental growth conditions [24]. Even for malignant cases of lymphoma, a study reported an average tumor doubling time of around 29 days [25]. These clinical reports confirm that physiological cell growth is extremely slower when comparing in vitro cell doubling times in different cancer cell types [26, 27]. Although much of these growth variations were attributed to cellular senescence, apoptosis, and necrosis, metabolic reprogramming is now believed to play a pivotal role [5, 6]. Given such a stark difference in growth kinetics, alternative cell culture conditions may prove more accurate representations of in vivo metabolic activity.

Evidence continues to compound that a 3D culture may be a better representation of in vivo cell growth, migration, and drug diffusion [28–30]. Several reports have evaluated DLBCL cell lines and primary cells cultured in 3D models (i.e., biomaterial scaffolds, hydrogels, and spheroids) [31–37], though none has evaluated metabolic reprogramming. Recent studies on colorectal cancer [38] and epithelial cells [39] have corroborated that a 3D culture can impact metabolism giving credence to the likelihood that a 3D culture may alter DLBCL metabolism as well.

In this study, we analyzed DLBCL cells in 2D or 3D in vitro environments and measured cell proliferation, drug activity, and metabolic functions in the context of developing physiologically appropriate assay as compared to patient tumor tissue. We found that DLBCL cells exhibit controlled proliferation and metabolic activity as well as retain sensitivity to CHOP in a 3D environment. Additionally, DLBCL cells cultured in 3D showed a metabolite trend inclining towards physiological relevance when compared to patient DLBCL tissue samples.

## Methods

### DLBCL cell lines

DLBCL cells, SUDHL-10 (parental), SUDHL-10 RR (rituximab drug resistant), and SUDHL-10 OR (obinutuzumab drug resistant), were maintained in RPMI 1640 media (ATCC 30-2001^TM^) supplemented with 10% fetal bovine serum (FBS) and 1% antibiotic-antimycotic solution (Fisher Scientific, Boston, MA) at 37 °C in a humidified atmosphere with 5% CO_2_. The biological characteristics of these lymphoma cell lines are outlined in [40], and these cells were utilized for experiments performed with 2D and 3D cell cultures. Frozen DLBCL patient tissue samples were isolated from IRB-exempt discarded tissue specimens of patients with diffuse large B-cell lymphoma (DLBCL) obtained through Rutgers CINJ Biospecimen Repository.

### 3D cell culture and recovery

VitroGel® (TheWell Bioscience, NJ, USA) — a xeno-free functional hydrogel matrix — was used for the generation of the 3D cell culture. Briefly, the cell suspensions of required cell density were reconstituted in 30% FBS consisting VitroGel solution® (gel to cell ratio 2:1) by gentle pipetting. The cell-hydrogel mixture was then transferred to cell culture plates allowed to evenly distribute and form hydrogel at room temperature for 15 min followed by the addition of growth media equal to the corresponding hydrogel volume. The prepared plates were returned to a 37 °C incubator and maintained in a humidified atmosphere with 5% CO_2_, and 70% medium was refreshed every 48 h.

**Fig. 1:**
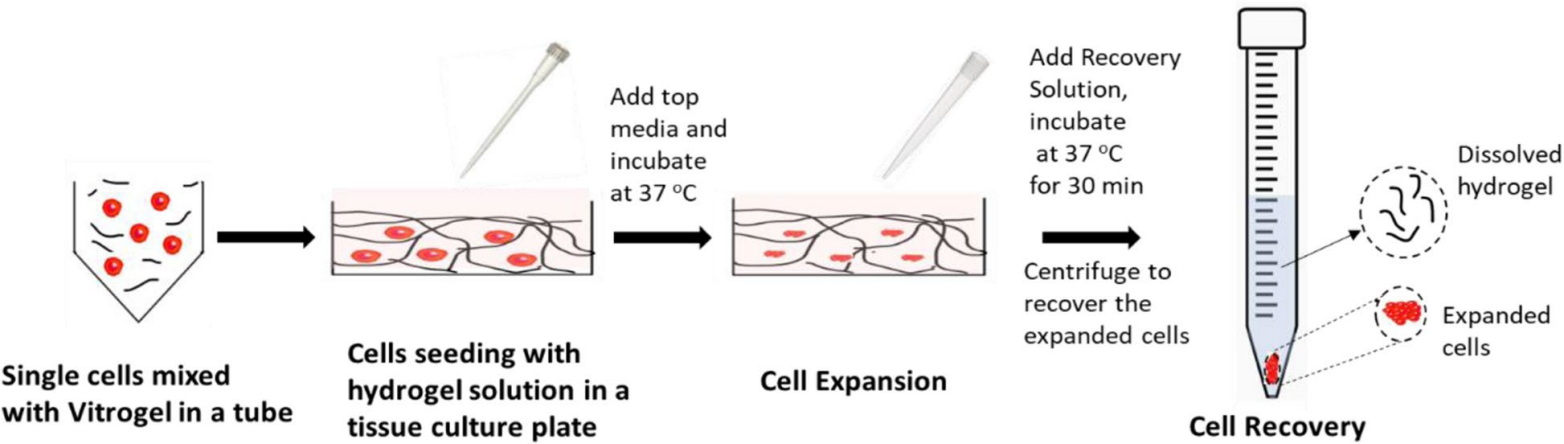
Schematic representation of expansion and recovery of DLBCL cell lines in 3D hydrogel “VitroGel®”

For the recovery of cells from hydrogel, a non-enzymatic VitroGel recovery solution was utilized following manufacturer recommendations. Briefly, prewarmed (37 °C), recovery solution was added to hydrogel, gently mixed for disrupting the hydrogel, followed by 37 °C incubation in water bath for 20 min with intermittent mixing. Hydrogel-released cells were recovered by centrifugation at 123 ×g for 7 min and stored or utilized for downstream applications, as summarized in Fig. 1.

### Cell proliferation assay

Cell proliferation was quantified using automated NucleoCounter NC-202^TM^. For 3D expansion of lymphoma cell lines, 5.0 × 10^4^ cells/well were seeded in a 48-well plate consisting of 150 μL of hydrogel, topped up with an additional 150 μL of growth media or 2D cell culture (without hydrogel), and incubated at 37 °C in a humidified atmosphere with 5% CO_2_. Cells recovered using a recovery solution for hydrogel or an aliquot from 2D was used for cell count performed each day for 5 days with NucleoCounter NC-202.

### Flow cytometric cell cycle analysis

SUDHL-10 cells were seeded at a seeding density of 6 × 10^5^ cells/well in a 24-well plate consisting of 300 μL of hydrogel, topped up with an additional 300 μL of growth media or 2D cell culture (without hydrogel), and incubated at 37 °C in a humidified atmosphere with 5% CO_2_. Cells recovered using a recovery solution for hydrogel or from 2D at day 1 and day 5 were washed with phosphate-buffered saline (PBS), fixed in precooled 70% ethanol, and stored at 4 °C. Finally, after washing with PBS, the cells were stained with FxCycle™ PI/RNase Staining Solution (PI) (Invitrogen, MA, USA) and analyzed by an FC 500 flow cytometer (Beckman Coulter, IN, USA). Data were analyzed using FlowJo (BD Biosciences, CA, USA). All experiments were performed in experimental triplicates, with error bars represented in data indicating standard deviations, and statistically significant differences were determined by a 2-tailed, *t*-test, comparing 2D grown cells with 3D conditions.

### Apoptosis assay

SUDHL10 cells were seeded at a seeding density of 2 × 10^5^ cells/well in a 24-well plate for 2D and 3D cultures as per the method described in cell cycle analysis. After 48 h of incubation, cells were recovered, washed with PBS, and stained using an annexin V apoptosis detection kit (Thermo Fisher Scientific, USA). Stained cells were analyzed by FC 500 flow cytometer. Analysis of stained cells can distinguish cells into four groups, namely viable (annexin V^−^ PI^−^), early apoptotic (annexin V^+^ PI^−^), late apoptotic (annexin V^+^ PI^+^), and necrotic (annexin V^−^ PI^+^) cells.

### In vitro drug sensitivity assay

Cells cultured in 2D or 3D were allowed to grow for 24 h followed by the addition of CHOP, cyclophosphamide, doxorubicin, vincristine, and prednisone, at a clinical ratio of 80/5.5/0.16/11.1 [41] at concentrations ranging from 0 to 1280 ng/mL. After 48 h of drug exposure, cell survival was determined using the Cell Counting Kit-8 assay (CCK-8; Sigma, USA) following the manufacturer-supplied instructions. Briefly, 10 μL of CCK-8 reagent was added to each well and plates were incubated for 4 h at 37 °C followed by spectrophotometric quantification by measuring absorbance at 450 nm using an automatic plate reader, Varioskan LUX multimode microplate reader (Life Technologies Corporation, NY, USA).

### Metabolic profiling of DLBCL cell lines cultured in 2D vs 3D and frozen DLBCL patient tissue samples

2 × 10^5^ cells were seeded in 48-well plates, after 48 h of incubation cell culture supernatant and whole cell lysates were harvested for metabolic profiling analysis. Glucose, glutamine, and lactate levels from cell culture supernatant were analyzed using Cedex Bioanalyzer (Roche Diagnostics Corp, NC, USA). Cell pellets were lysed using (40:40:10) methanol, acetonitrile, and water containing 0.5% ice-cold formic acid. The lysates were transferred to a dry ice methanol bath and kept for 5 min before neutralizing with 15% sodium bicarbonate. Neutralized lysates were centrifuged at 13000 ×g for 10 min, and supernatants were collected and stored at −80°C for analysis by mass spectrometry. For 3D hydrogel expanded cell lysate preparation, an additional filtration using a 10-kDa centrifugal filter at 10,000 ×g for 10 min at 4°C was performed to prevent hydrogel carry over into lysate. LC-MS analysis of cellular metabolites was performed on the Q Exactive PLUS hybrid quadrupole-orbitrap mass spectrometer (Thermo Scientific, USA) coupled to hydrophilic interaction liquid chromatography (HILIC). Metabolite features were extracted in MAVEN with the labeled isotope specified and a mass accuracy window of 5 ppm [42]. The ^13^C isotope natural abundance and impurity of labeled substrate were corrected using AccuCor written in R [43]. The corrected ion counts normalized based on the cell number. For isotope tracer labeling, d-glucose-^13^C_6_ (Cambridge Isotope Laboratories, MA, USA) substituted in glucose, pyruvate, and bicarbonate-free RPMI-1640 (Sigma Aldrich, MO, USA) consisting of 10% dialyzed FBS.

Frozen DLBCL patient tissue samples were collected under the IRB-exempt protocol. Intracellular metabolites were extracted and analyzed using LC-MS. Four selected metabolites namely glycerol 3-phosphate, inosine monophosphate (IMP), glyceraldehyde 3-phosphate, and ribulose 5-phosphate were analyzed at four different concentrations (2000, 200, 20, and 2 μM) along with DLBCL frozen tissue extracts and SUDHL-10 extracts from 2D and 3D grown for 48 h. Final obtained concentrations were normalized against milligrams of proteins obtained from each of these samples. Protein concentrations were measured using Protein Assay Reagent, Biorad, CA, USA.

### Statistical analysis

A Student *t*-test was performed using GraphPad Prism and Microsoft Excel software. A *p*-value of less than 0.05 was considered significant. All experiments described under the “Methods” section were performed in triplicates with values reported as mean ± SD.

## Results

### DLBCL cells have attenuated growth and upregulated apoptosis in a 3D culture

**Fig. 2:**
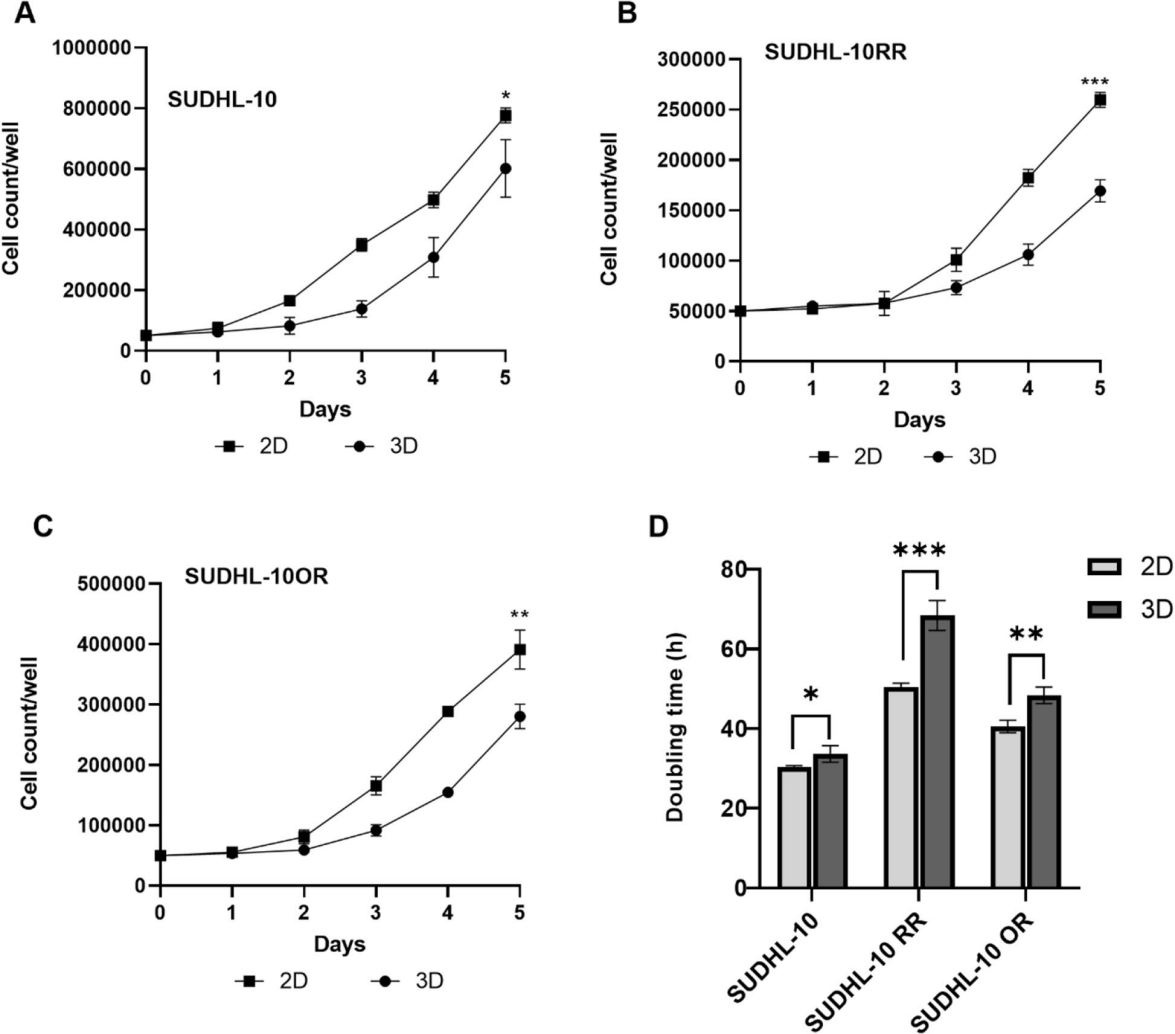
Cell proliferation profile of **A** SUDHL-10, **B** SUDHL-10 RR, and **C** SUDHL-10 OR and **D** doubling time variations of all three cell lines in 2D and 3D. Values indicate mean± SD. Experiments were done in triplicate. A 2-tailed *t*-test was used to determine significance between 2D and 3D denoted by *** (*p*<0.0001), ** (*p*<0.005), and * (*p*<0.05)

Initial studies first evaluated the growth kinetics of DLBCL cell lines (parental and resistant) when cultured in 2D and 3D conditions. 3D scaffold conditions promoted cell expansion, albeit at a significantly slower rate than a conventional 2D culture (Fig. 2A–C). In 3D conditions, the SUDHL-10 parental cell line grew faster than resistant cell lines with a 12-fold increase in growth in comparison to a 4-fold and 6-fold increase for SUDHL-10 RR and SUDHL-10 OR, respectively. All three cell lines exhibited a more constrained cell proliferation in 3D with a significantly high doubling time compared to 2D (Fig. 2D).

To further understand the causes of slowed proliferation in 3D, we monitored cell death for SUDHL-10 using flow cytometry after 48 h of growth. In 2D, ~90% of cells were viable after 2 days as opposed to ~ 80% viable cells in 3D. Of the non-viable cells, there was a slight increase in necrosis with a more significant impact on early and late apoptotic populations in 3D compared to 2D culture (Fig. S1A). These results suggest that the 3D culture can instigate apoptotic pathways, not dissimilar from native in vivo conditions.

### DLBCL cells grown in 3D display significantly reduced cell cycle progression

**Fig. 3:**
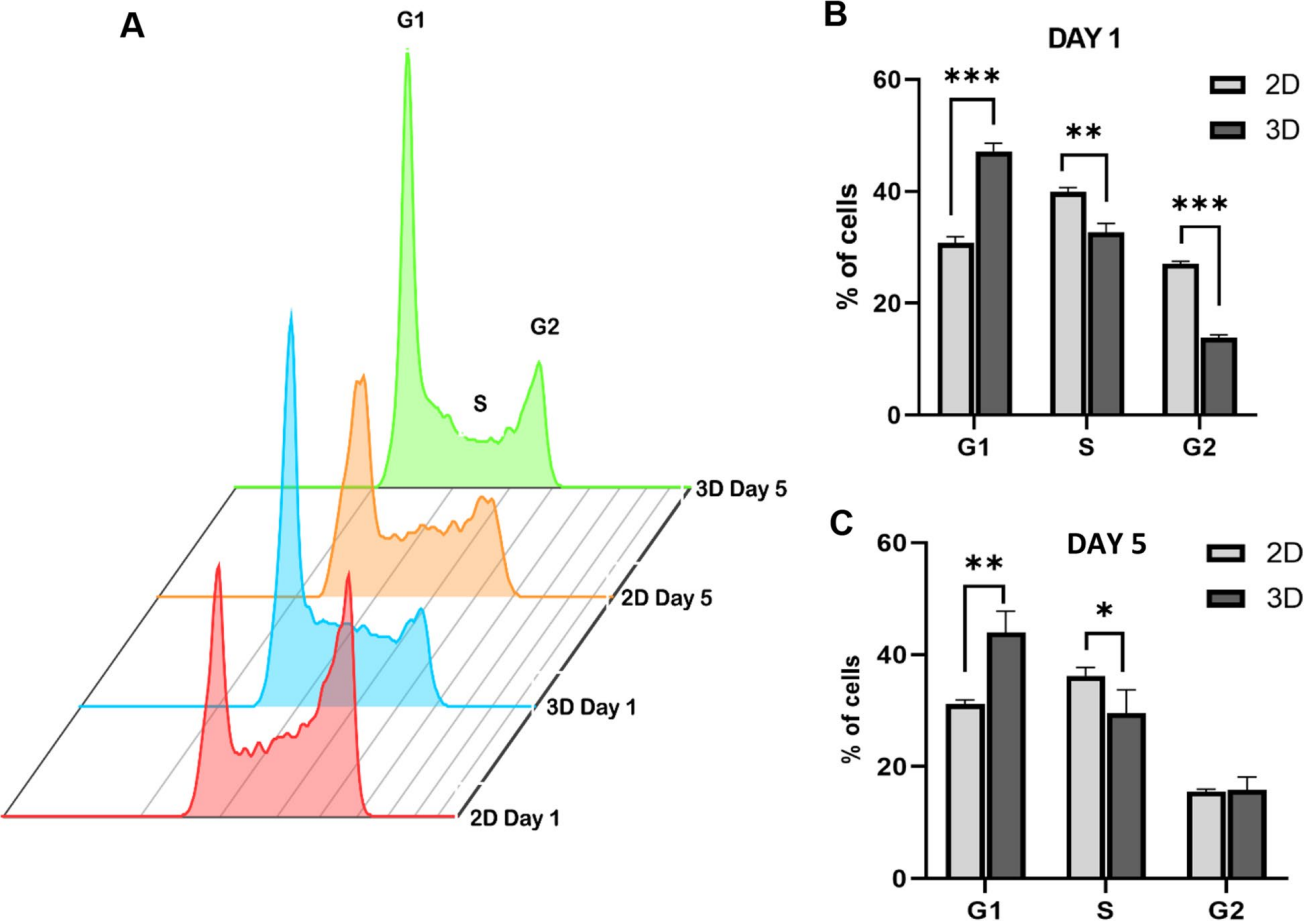
Quantification of SUDHL-10 cell cycle analysis performed using flow cytometry from three independent experiments **A** overlay plot, day 1 (**B**) and day 5 (**C**). Values indicate mean ± SD. Experiments were done in triplicate. A 2-tailed *t*-test was used to determine significance between 2D and 3D denoted by *** (*p*<0.0001), ** (*p*<0.005) and * (*p*<0.05)

An alternative mechanism of slowed growth could be that there was differential cell cycling of DLBCL cells in 2D and 3D culture formats. PI incorporation was used to determine the cell cycle distribution of cells grown in 2D or 3D conditions. SUDHL-10 cells showed a significant increase (~20% more) of the proportion of cells in the G1 phase in 3D when compared to 2D (Fig. 3A). Conversely, 2D conditions elicited a higher accumulation of cells in S and G2 phases (Fig. 3A). These cell cycle results were observed 1 day after stabilizing in culture. By day 5 in culture, the G1/S cycling behavior of 3D cells was still observed though the G2 phase was equally proportionate in 2D and 3D cultures (Fig. 3B). Taken together, we conclude that SUDHL-10 cells experience a significant delay in G1/S transition under 3D.

### DLBCL cell line response to CHOP in 2D and 3D

**Fig. 4:**
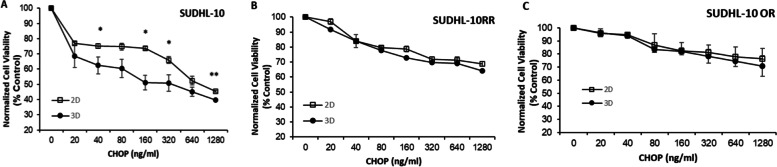
Effect of CHOP on the viability of SUDHL-10 (**A**), SUDHL-10 RR (**B**), and SUDHL-10 OR (**C**) in 2D and 3D determined by CCK-8 assay. Cells were allowed to grow for 24 h and then treated with increasing concentrations of CHOP for 48 h. Error bars represent the standard deviation of means from replicates consisting of three independent experiments. A 2-tailed *t*-test was used to determine significance ** (*p*<0.005) and * (*p*<0.05) when compared to 2D

A multi-drug cocktail consisting of cyclophosphamide, hydroxydaunorubicin, vincristine, and prednisone (referred to as CHOP) is the first-line therapy for DLBCL patients. A clinically relevant dose range of CHOP (20–1280 ng/mL) was tested on parental and drug-resistant DLBCL cell lines to evaluate drug sensitivity across 2D and 3D conditions. Firstly, results indicated significant differences in the cytotoxic responses of CHOP for the parental line SUDHL10 when compared to resistant lines (SUDHL-10 RR, SUDHL-10 OR; Fig. 4A–C) in both 2D and 3D culture conditions. Both SUDHL-10RR and SUDHL-10 OR showed higher resistance with > 65% viability compared to 40% viable SUDHL-10 cells after 48h of CHOP treatment. To our knowledge, these studies are the first to report CHOP resistance in these cell lines that already have conferred resistant to B-cell depleting antibodies.

When comparing CHOP sensitivity in an individual cell line across 2D and 3D formats, there were selective alterations only in the parental SUDHL-10 line. The inhibitory concentration (IC_50_) values for CHOP in SUDHL-10 cultured in a 3D matrix (320 ng/mL) and 2D (640 ng/mL) suggest that cells grown in 3D exhibit higher drug sensitivity (Fig. 4A; *p*=0.03). On the other hand, rituximab-resistant SUDHL-10 RR (Fig. 4B) and obinutuzumab-resistant SUDHL-10 OR (Fig. 4C) showed resistance against CHOP, independent of the culture format, and never reached an IC_50_ threshold within tested CHOP concentration range.

### Comparing extracellular and intracellular metabolite levels in 2D vs 3D

**Fig. 5:**
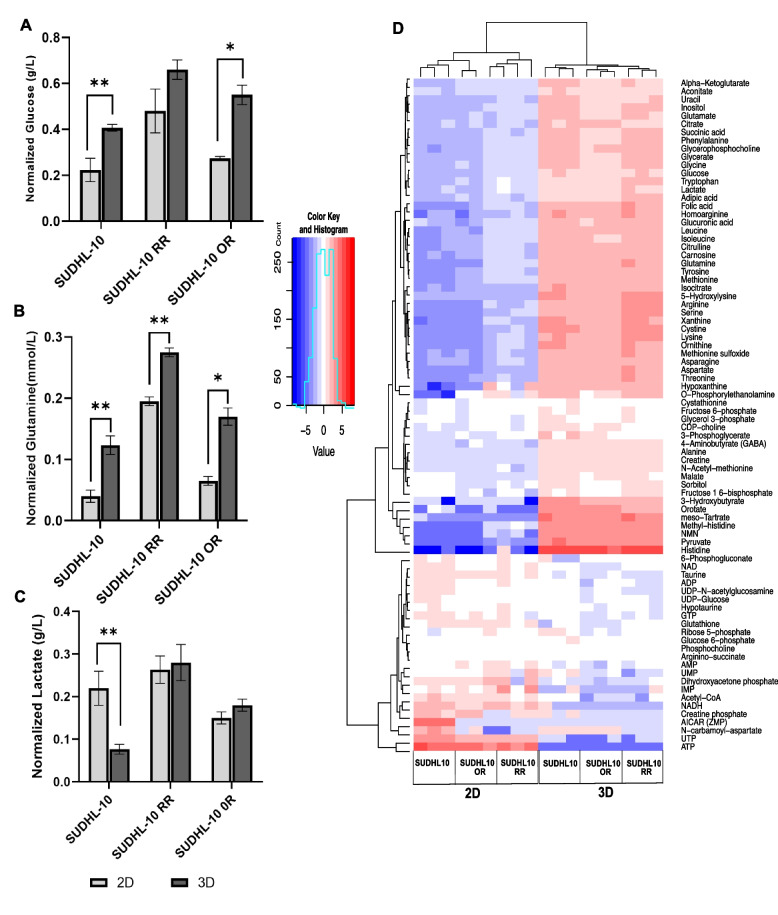
Metabolic profiling of DLBCL cell lines in 2D vs 3D. Analysis of extracellular metabolites using Cedex Bioanalyzer: **A** glucose, **B** glutamine, and **C** lactate. Data is normalized to the ratio of starting to final cell densities. Data obtained from *n*=3 repeats, values indicate mean± SD. A 2-tailed *t*-test was used to determine significance ** (*p*<0.005) and * (*p*<0.05). **D** LC-MS analysis of intracellular metabolite exhibiting differential pattern between 2D vs 3D

A 3D matrix imparts structural constraints similar to natural tissues, limiting cell proliferation and access to metabolic substrates. Considering that DLBCL cells exhibited reduced proliferation with slower cell cycle activity under a 3D culture, we chose to determine whether access to metabolic fuel supply becomes a growth-limiting barrier under 3D. Metabolite profiling of extracellular medium showed significant differences in extracellular levels of glucose and glutamine concentrations between 2D vs 3D (Fig. 5A). Higher amounts of glucose and glutamine were accumulated in the extracellular medium of 3D cultured cells suggesting lesser utilization. SUDHL-10 showed 47% less utilization of glucose and 66% of glutamine in 3D when compared to 2D. Similarly, reduced utilization of glucose and glutamine was also observed for SUDHL-10 RR and SUDHL-10 OR cell lines. Owing to higher metabolic activity in 2D, increased secretions of lactate (2.7-fold) were also observed in SUDHL-10 when compared to 3D, though not much variation was seen for drug-resistant cell lines (Fig. 5A).

Since extracellular concentrations of glucose or glutamine were consistently higher in SUDHL-10, SUDH-10 RR, or SUDHL-10 OR cells, further investigation focused on the intracellular metabolic activity and fluxes in cultured cells using LC-MS. Various polar metabolites associated with glycolysis, Krebs cycle, nucleotide metabolism, amino acid metabolism, urea cycle, and polyamines were identified as affected, shown in Fig. 5B. The heatmap representing metabolite pools of SUDHL-10 parental and resistant cells grown in 2D vs 3D segregated into two distinct clusters. The first cluster consisted metabolites predominantly associated with glycolysis, Krebs cycle, and amino acid metabolism that were elevated in 3D when compared to 2D. The second cluster comprised metabolites related to rate-limiting steps of the oxidative branch of the pentose phosphate pathway and nucleotide biosynthesis (which includes glucose-6-phosphate, ribose-5-phosphate, inosine monophosphate, carbamoyl aspartate, UTP, and ATP). This cluster pool size was observed to be decreased in all three DLBCL cell lines grown in 3D (blue color, Fig. 5B). These results represent a possible slowdown in nucleic acid synthesis activity associated with proliferative function resulting from metabolic diversion towards glycolysis, Krebs cycle, and amino-acid metabolism in SUDHL10 cells cultured under 3D.

### Metabolic flux analysis showed the 3D culture induces glycolysis and lower nucleotide synthesis

**Fig. 6:**
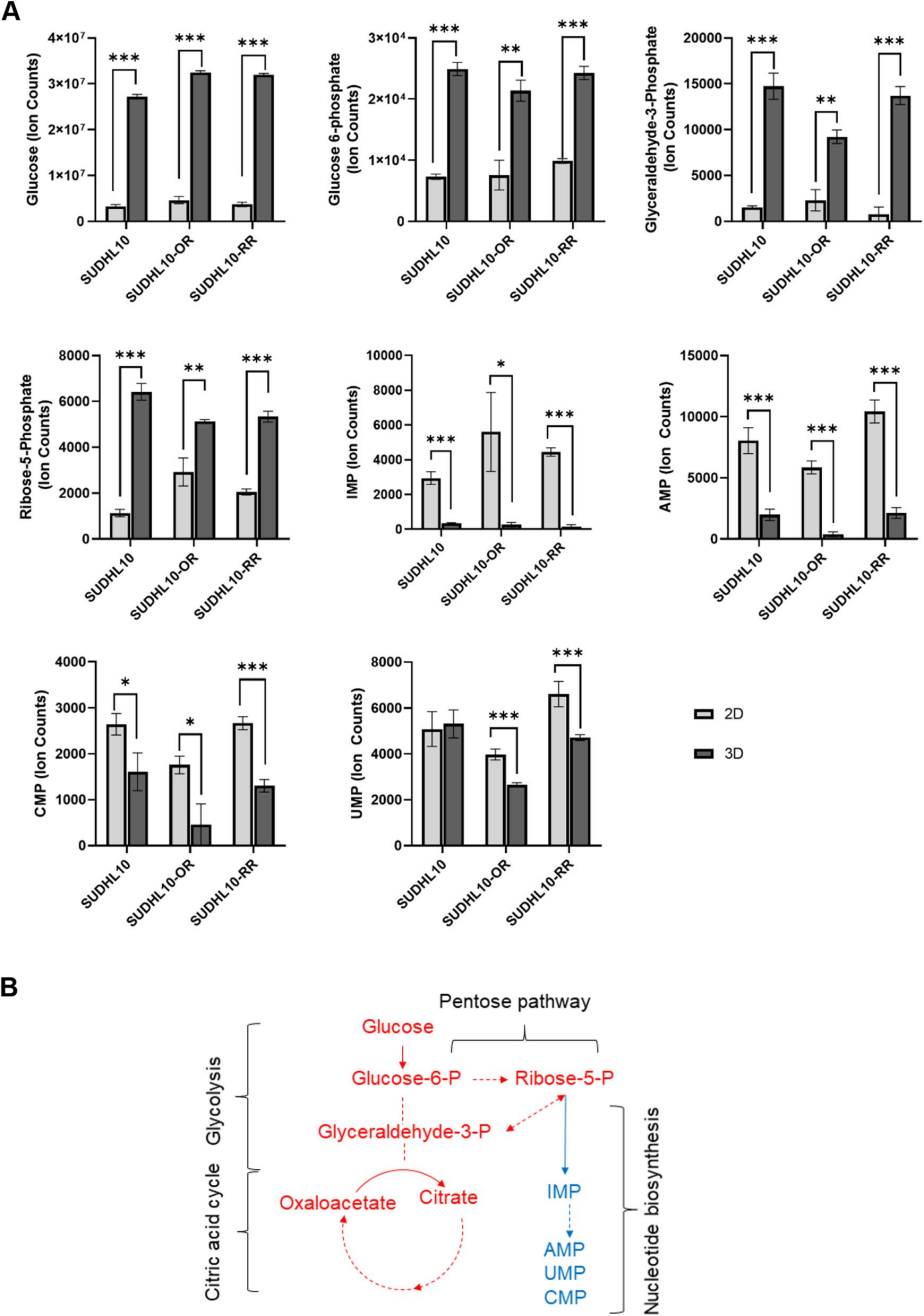
3D grown DLBCL cell lines show decreased nucleotide metabolism. **A** Total pool size of metabolites in SUDHL10, SUDHL10OR, and SUDHL10RR cells cultured in 2D vs 3D conditions for 48 h. **B** Summary of metabolic pathway changes represented by labels indicated in red (increased) or blue (decreased) in 3D cultured cells compared to 2D

Metabolic flux analysis was then performed. C^13^-glucose labeling and isotope tracer studies revealed glucose uptake is significantly elevated in SUDHL10 parental or resistant cells when cultured in 3D (Fig. 6), as indicated by total pool size increases (Fig. 6A) and fractional labeling pattern (Fig. S3A-B). These patterns correlated with the observation of elevated concentrations of glucose-6-phosphate in total intracellular metabolite pool analysis of SUDHL10 parental or resistant cells cultured in 3D (Fig. 5B). Furthermore, increased total pool sizes of intracellular glyceraldehyde-3-phosphate and ribose 5-phosphate indicate that glycolytic activity is elevated in 3D. On the other hand, reduced total pool size of nucleotide biosynthesis precursor IMP and accompanying products including AMP, CMP, and UMP was observed in DLBCL cells indicating that reduced nucleotide biosynthesis is occurring in 3D (Fig. 6A). Together, these observations suggest a redirection of glucose carbon from nucleotide metabolism towards glucose oxidation by glycolysis in DLBCL cells cultured in 3D (Fig. 6B). Overall, increased glycolysis and diminished nucleotide biosynthesis are commonly observed in DLBCL cells grown in 3D, and further 3D matrix did not impair glucose uptake but rather altered metabolism which perfectly correlates with the diminished proliferative activity in DLBCL cells grown in 3D.

### Comparison of metabolite concentrations in 2D/3D cultured cells and tumor tissue

**Fig. 7:**
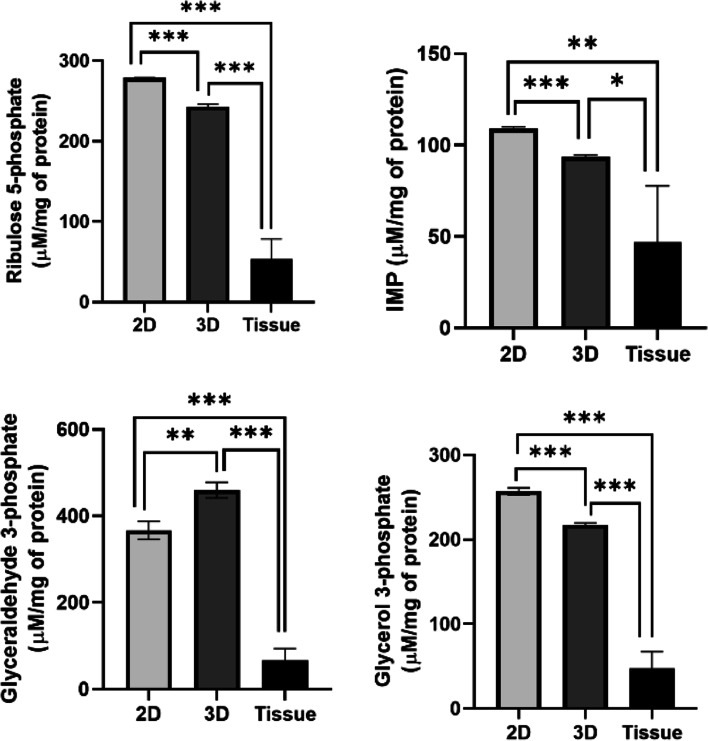
Comparison of DLBCL metabolite concentrations by quantitative mass spectrometry. Bar graphs represent the concentration of individual metabolites normalized per milligram of total protein (*y*-axis), in DLBCL tumors (*n*=10) and triplicates of SUDHL-10 cells cultured in 2D or 3D (*x*-axis). Error bars represent the standard deviation of means by replicates. Significant differences in metabolite concentration between the sample groups are denoted by *** (*p*<0.0001), ** (*p*<0.005), and * (*p*<0.05)

Finally, considering that the metabolic variations were observed in cells cultured in 2D and 3D cells, our next goal was to determine which of these metabolic profile trends closely match with that of natural DLBCL tumor tissues. To do this, based on differential glycolytic flux pattern observed in 3D cultured DLBCL cells, absolute concentrations of four metabolites which include ribulose 5-phosphate, inosine monophosphate (IMP), glyceraldehyde 3-phosphate, and glycerol 3-phosphate, extracted from SUDHL-10 cells cultured in 3D or 2D, were compared against fresh snap frozen DLBCL patient tumor tissues (*n*=10). The metabolite concentrations were normalized per milligram of protein. The results from 3D cultured cells indicated that metabolite levels were trending to decrease towards tissue concentrations, in SUDHL-10 cells maintained in 3D for as little as 48 h. Altogether, the metabolite concentrations of (*n*=10) natural tumors are certainly lower than that of cultured cells maintained in 2D or 3D; however, with robust glycolytic activity and reduced nucleotide metabolism, cells under 3D seem gradually shifting towards natural concentrations of tissue metabolites (Fig. 7).

## Discussion

In recent years, 3D lymphoma models have been used increasingly to provide insight into the tumor microenvironment, therapeutic potential, and resistances [44–47]. Even though most of the cancer reports on in vitro drug discovery and testing are performed in 2D [21], three-dimensional (3D) culture platforms are now gaining place on the frontiers of oncology research, mainly because of their presumed simulation to in vivo cell microenvironment and reliability of mimicking disease state [48, 49].

This study evaluated if 2D or 3D culture conditions reflected physiologically relevant metabolism of patient lymphoma tumor tissue. Several natural, synthetic, and semi-synthetic bioinspired hydrogels [50, 51] have been developed for models of breast cancer [52, 53], cervical cancer [54], sarcoma [55, 56], and prostate cancer [57]. With a panoply of materials available, we chose a xeno-free, polysaccharide-based functional matrix for 3D expansion of lymphoma cells (Fig. 1). This material was chosen for its practicality of use in future clinical research; VitroGel is commercially available and easy to polymerize and has a non-enzymatic, fast recovery of 3D cultured cells. Several variations of Vitrogel are available and, upon screening, it was found that all matrices slowed the growth of a B cell line, DS-1, when compared to 2D (Fig. S2). VitroGel has been previously reported to study various cancer types including pancreatic cancer [58], melanoma [59], breast cancer [60, 61], non-small cell lung cancer [62], and osteosarcoma [63]; however, its application in DLBCL investigations is yet to be explored. Though this relatively simple, feasible, and reproducible 3D model led to a more clinically relevant phenotype of DLBCL cells, it lacks the interaction of cancer cells with other cell types comprising tumor microenvironment, such as stromal cells and immune cells. Further co-culture studies incorporating these cell types will be useful to improve the relevance of this model as a mimic of in vivo tumors.

Cell proliferation studies showed that VitroGel exerted a significant slowing of growth in 3D culture. These results were interpreted as a matrix-mediated constraint of cell growth rather than a cytotoxic effect of the scaffold given a marginal increase in metrics of necrosis and apoptosis (Fig. S1). We also observed a similar impact of matrix slowing growth of another B cell line, DS-1, when compared to 2D (Fig. S2). Indeed, our results aligned with diminished growth properties in 3D observed with several other cancer cell types [64, 65] including colorectal cancer [66], endometrial cancer [67], head and neck cancer [68], and breast cancer [69]. In contrast, some cancer cell types showed opposite proliferation rates with cells growing faster in 3D than in 2D [33, 70, 71]. For example, breast cancer cells, JIMT1, grew faster in Matrigel than in 2D. Interestingly, the same cell line exhibited a 7.2-fold slower growth rate when cultured on a synthetic poly (2-hydroxyethylmethacrylate) 3D scaffold, which suggests that cell proliferation rate is related to the type of matrix in which the cells are cultured. Furthermore, the mechanical properties of these matrices are also a major signaling factor via mechano-transduction that can influence cell growth, arrest, or apoptosis. Several reports have established the role of integrins [72, 73] and signaling molecules [74] in the survival of lymphomas. Further investigation on the effect of 3D matrix on expression levels of various signaling molecules like integrin-linked kinase, mitogen-activated protein kinase (ERK), and phosphoinositide 3-kinase (PI3-K) impacting the activity of anti-apoptotic BCL-2 family proteins, NF-kB, and AKT (forkhead and IAP protein regulators) would be interesting and might provide further insights on cross-talk between lymphoma cells and extracellular matrix [75].

Results from cell cycle analysis of asynchronously grown SUDHL-10 cells show that cell populations in the G1 phase were increased in 3D cell cultures (Fig. 3). A recent study highlighted variations in cell cycle between 2D and 3D cultured HeLa cell lines and emphasized the influence of collagen 3D matrix environment on cell cycle and cell viability with cells arrested in the G1/S phase in 3D [76]. Although reduced levels of secreted autocrine growth factors could limit the cellular growth in 3D, these effects were found to be mitigated by altering the cell densities [77]. By day 5, the percentage of G2 cells in 2D became similar to those in 3D which could be attributed to cells that have cycled through and divided within 5 days while 3D cells did not change their entry into G2 during this time. These results collectively suggest that 3D scaffolds induced cell cycle arrest consistent with a slower growth rate. Further investigations analyzing differences in expression levels of cell cycle regulatory genes including cyclin, cyclin-dependent kinases (CDK), and CDK inhibitors could provide a clear understanding of the role of matrix on lymphoma cell growth.

Along with variations in cell cycle, there was a marginal, though significant, difference in early/late apoptotic rates in the 3D culture when compared to the 2D culture of SUDHL-10 cells. The convention thought is that reduced apoptosis is a hallmark of carcinogenesis [78] and has been corroborated in several cancer reports where resistance to apoptosis in 3D culture has been observed [34, 38, 79, 80]. Yet, paradoxically, there are also several reports that link increased apoptosis to aggressive disease in multiple malignancies [81–86]. In fact, significant cell loss is reported in aggressive tumors [87]. Apoptosis signals may be critical for native tumor cell function and we, therefore, consider it an important feature to capture within in vitro model systems. Polysaccharides found within VitroGel hydrogel are hypothesized to induce this apoptotic effect on DLBCL cells. An algal sulfated polysaccharide extract suppressed cell proliferation of breast cancer cell line MDA-MB-231 and arrested them at the G1 phase. It was also shown to trigger apoptosis through enhanced expression of *Bax* and inhibition of Bcl-2 protein [88]. Other studies revealed that, Fucoidan, a sulfated polysaccharide from brown seaweed, induced apoptosis and inhibited angiogenesis in 4T1 mouse breast cancer cells and in BALB/c mice bearing breast cancer [89]. These studies indicate a possible involvement of polysaccharides in cell cycle arrest and apoptosis through enhanced expression of *Bax* and inhibition of Bcl-2 protein.

Drug sensitivity between 3D and 2D cultures of other tumors including lymphoma has been reported with mixed results; some report a 3D matrix imparting drug resistance [21, 34, 90, 91] while others show enhanced drug sensitivity [69, 92–94]. In our study, a comparison of CHOP sensitivity showed that the parental cell line (SUDHL-10) was more sensitive to CHOP in 3D. Several reasons could account for this observation. CHOP, or components of it, could have been concentrated and more locally bioavailable in a matrix rather than in a free-floating 2D culture. Increased sensitivity of parental cell line could be a synergistic effect of 3D matrix impact and CHOP — a chemotherapy well known for inducing cell cycle arrest and apoptosis leading to cell death [95]. Being cultured in 3D or in the presence of a polysaccharide-based matrix could also change the expression and/or activation levels of various signaling proteins like focal adhesion kinase (FAK) that regulate cell proliferation and survival [96]. Such variations could also impart matrix-dependent drug sensitivity to cells in 3D. Interestingly, the inherent resistance to CHOP by SUDHL-10 RR and SUDHL-10 OR cells were retained and not induced by 3D matrix conditions. It is well reported that these resistant cell lines often demonstrate a lack of sensitivity to multiple cytotoxic chemotherapeutic agents [97, 98]. Our observations indicate that the impact of the 3D matrix in sensitizing a parental line was independent of the resistance of CHOP by rituximab- and obinutuzumab-resistant DLBCL cell lines.

The metabolism of DLBCL cells was also characterized in 2D and 3D environments. In vitro, 2D culture condition generally exposes cells to supraphysiological levels of nutrients which could result in metabolic artifacts. In contrast, the 3D architecture act as a barrier slowing the access to extracellular metabolites and potentially concentrating cell-secreted forms in a physiologically relevant manner [29]. Analysis and profiling of extracellular metabolites including glucose and glutamine indicated the presence of higher levels of these metabolites in 3D (Fig. 5A, B). Glucose is the primary carbon source for anabolic reactions and energy production in cells, whereas glutamine uptake essentially supports the synthesis of purine/pyrimidine and NADPH and helps in maintaining the cellular redox balance [99, 100]. With SUDHL-10 RR and SUDHL-10 OR that tend to proliferate much slower compared to SUHDL-10, we noted the presence of higher concentrations of unspent glucose and glutamine levels in the medium. Similarly, increased lactate presence was detected in the media of DLBCL cells cultured in 2D or 3D. While lactate secretion occurs via the Warburg effect and is generally associated with proliferative activity [101–103], increased lactate secretion in 2D was found to correlate with a higher proliferation of SUDHL-10 (Fig. 5C). Lactate and lactate dehydrogenase (LDH) are commonly used as clinical markers of tumor burden and progression and as indicators of responses to therapies in lymphoma [104, 105]. Therefore, increased levels of extracellular lactate simply reflect higher amounts of cell numbers present in 2D. In contrast, substantial lactate accumulation was observed in 3D for resistant cell lines. Whether it is due to impaired MCT1 — a monocarboxylate transporter on resistant cells [106] — or an impact of the 3D matrix is a matter of further investigation. Further analysis of intracellular metabolites (Fig. 5D) showed an expansion in the intracellular pool sizes of key metabolites including pyruvate, lactate, glutamate, and amino acids in 3D. Future studies can explore functional implications of these metabolic changes that globally reflect the bioenergetics of cells in 2D vs. 3D, which our results suggest may be impacted. In contrast, a reduction in the pool sizes of metabolites associated with nucleotide biosynthesis (which includes IMP, AMP, UMP, Acetyl-CoA, and N-carbamoyl-aspartate) was also observed in 3D. Cell cycle analysis shows an elevated accumulation of cells in the G1 phase, indirectly indicating a slow down of DNA synthesis in cells grown in 3D when compared to 2D although DNA synthesis analysis would be warranted to further conclude this functional outcome. All these changes reflect an effect of accompanying increased glycolytic and citric acid metabolic activity observed in 3D. ^13^C isotope tracer metabolic profiling further confirmed occurrence of enhanced glucose uptake in DLBCL cells cultured under 3D as evident from increased pool size and fractional labeling patterns observed in Fig. 6 and Fig. S3A-B. Additionally, increased levels of glucose-6-phosphate (Fig. 6A) along with its decreased fractional labeling (Fig. S3) support enhanced glucose metabolic activity occurring in 3D. Interestingly, while the glycolytic activity was high in 3D compared to 2D, the fractional labeling pattern did not show much difference between 2D and 3D, confirming metabolic rates are similar in both 2D and 3D. Additionally, along with elevated glycolysis, reduced nucleotide metabolism was evident by the total pool analysis (Fig. 6A). Such variations could be attributed to the suppression of the rate-limiting step (ribose-5-phosphate to IMP) in nucleotide biosynthesis or its activation in 2D conditions. However, further experimentations will be required for confirmation. Similar metabolic variations with suppression of nucleotide metabolism accompanied by increased glycolytic activity were observed in our recent studies investigating the impact of fatty acid synthase (FASN) inhibition on the nucleotide metabolism in non-Hodgkin’s lymphoma [107]. Furthermore, with increased glycolysis and TCA, along with decreased de novo nucleotide biosynthesis, impairment of DNA/RNA synthesis and cell cycle was also observed [107]. Several other studies reported the importance of diverting glycolytic flux into the non-oxidative branch of the pentose phosphate pathway to generate ribose-5-phosphate which can then be utilized for nucleotide biosynthesis [108].

The differences in metabolic activity between DLBCL cells cultured in 2D and 3D environments were evident; benchmarking these in vitro functions to true clinical specimens was important to qualify one model as more clinically relevant. Considering that in vivo tissue metabolic activity is unlikely to be precisely comparable with in vitro culture of established cell lines, our initial goal in this study is limited to demonstrate that the trends of metabolites exhibited by DLBCL cells cultured in 3D are physiologically favorable. Therefore, we compared the absolute concentrations of select metabolites by quantitative mass spectrometry using DLBCL cells cultured in 2D or 3D against fresh snap frozen DLBCL tumors (Fig. 7). Metabolite concentrations of ribulose 5-phosphate, IMP, glyceraldehyde 3-phosphate, and glycerol 3-phosphate were considerably higher in 2D compared to DLBCL cells cultured in 3D. The mass of metabolites normalized to tissue protein from fresh tumors was significantly lower compared to cultured cells. These results suggest that decreased mass of metabolites in 3D may be a more favorable trend oriented towards physiological metabolic functions. There are several limitations to our study that restrict our conclusions without further investigation, particularly that tumor tissue is composed of a mixture of different cell types, only a fraction of which are malignant B cells, whereas our cell culture results are purely from a monoculture of a cancer cell line. Future analysis will benefit from malignant cells isolated from fresh tissue and comparing with long-term cultures of in vitro models maintained under physiologically relevant conditions. Conversely, model systems can build in complexity with additional cell types to attempt to match steady-state metabolite levels as an approach to developing more physiological bioassays.

## Conclusion

DLBCL cells cultured in 3D matrices have reduced proliferative activity and altered metabolism that is directionally consistent with in vivo tumor cell function. Continued development of 3D conditions that stabilize tumor cell function towards in vivo phenotype can prove useful in understanding and intervening cancer progression.

## Supplementary Information

**Additional file 1: Figure S1.** Apoptosis profile (A) Apoptosis analysis of SUDHL-10 cultured in 2D and 3D using flow-cytometry and (B) an overlay plot. Error bars represent standard deviation of means. Experiments done in triplicates. Significant differences between 2D and 3D are denoted by *** (*p*<0.0001), ** (*p*<0.005) and * (*p*<0.05).

**Additional file 2: Figure S2.** Cell proliferation profile (A) DS-1, (B) NHDF, (C) PBMC. Values indicate Mean± SD. Experiments were done in triplicate.

**Additional file 3: Figure S3.** ^13^C isotope tracer labeling to elucidate metabolic flux variations between 2D and 3D grown DLBCL cell lines. (A) C^13^ labeled total pool (B) C^13^ fractional labelling.

## Data Availability

All data and materials are fully described in the manuscript. A copy of all data analyzed is available from the corresponding author upon reasonable request.
