# Supplementary figures and images for: Scaffold-mediated switching of lymphoma metabolism in culture
Source: Cancer Metab. 2022 Oct 12;10:15. doi: 10.1186/s40170-022-00291-y (PMC9559005; doi:10.1186/s40170-022-00291-y)

Supplementary Figure S1

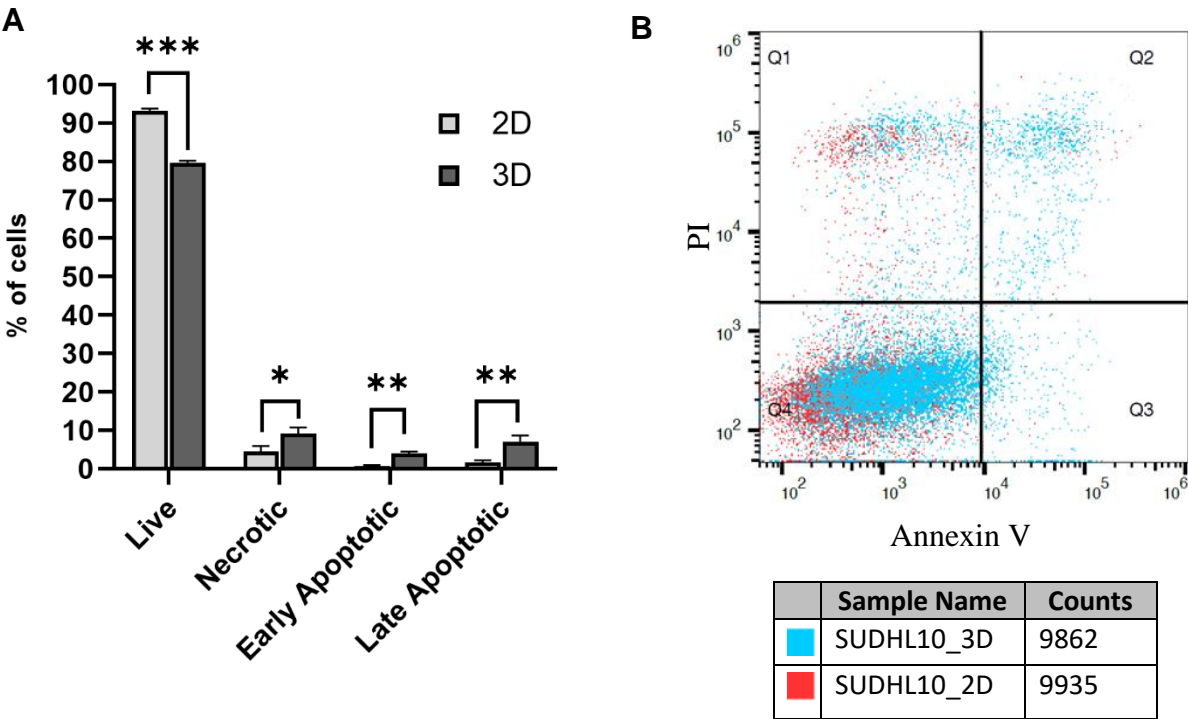

Supplement: Supplementary file 1 — Additional file 1: Figure S1. Apoptosis profile (A) Apoptosis analysis of SUDHL-10 cultured in 2D and 3D using flow-cytometry and (B) an overlay plot. Error bars represent standard deviation of means. Experiments done in triplicates. Significant differences between 2D and 3D are denoted by *** (p<0.0001), ** (p<0.005) and * (p<0.05). [file 40170_2022_291_MOESM1_ESM.pdf]

Supplementary Figure S2

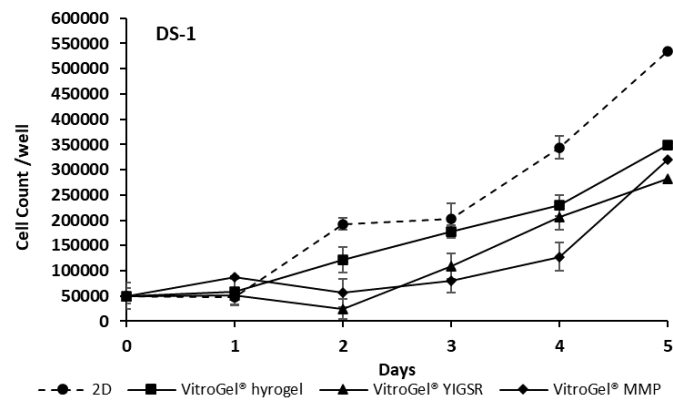

Supplement: Supplementary file 2 — Additional file 2: Figure S2. Cell proliferation profile (A) DS-1, (B) NHDF, (C) PBMC. Values indicate Mean± SD. Experiments were done in triplicate. [file 40170_2022_291_MOESM2_ESM.pdf]
