# Supplementary material for: Scaffold-mediated switching of lymphoma metabolism in culture
Source: Cancer Metab. 2022 Oct 12;10:15. doi: 10.1186/s40170-022-00291-y (PMC9559005; doi:10.1186/s40170-022-00291-y)

Supplementary Figure S3A: C^{13} Labeled Pool

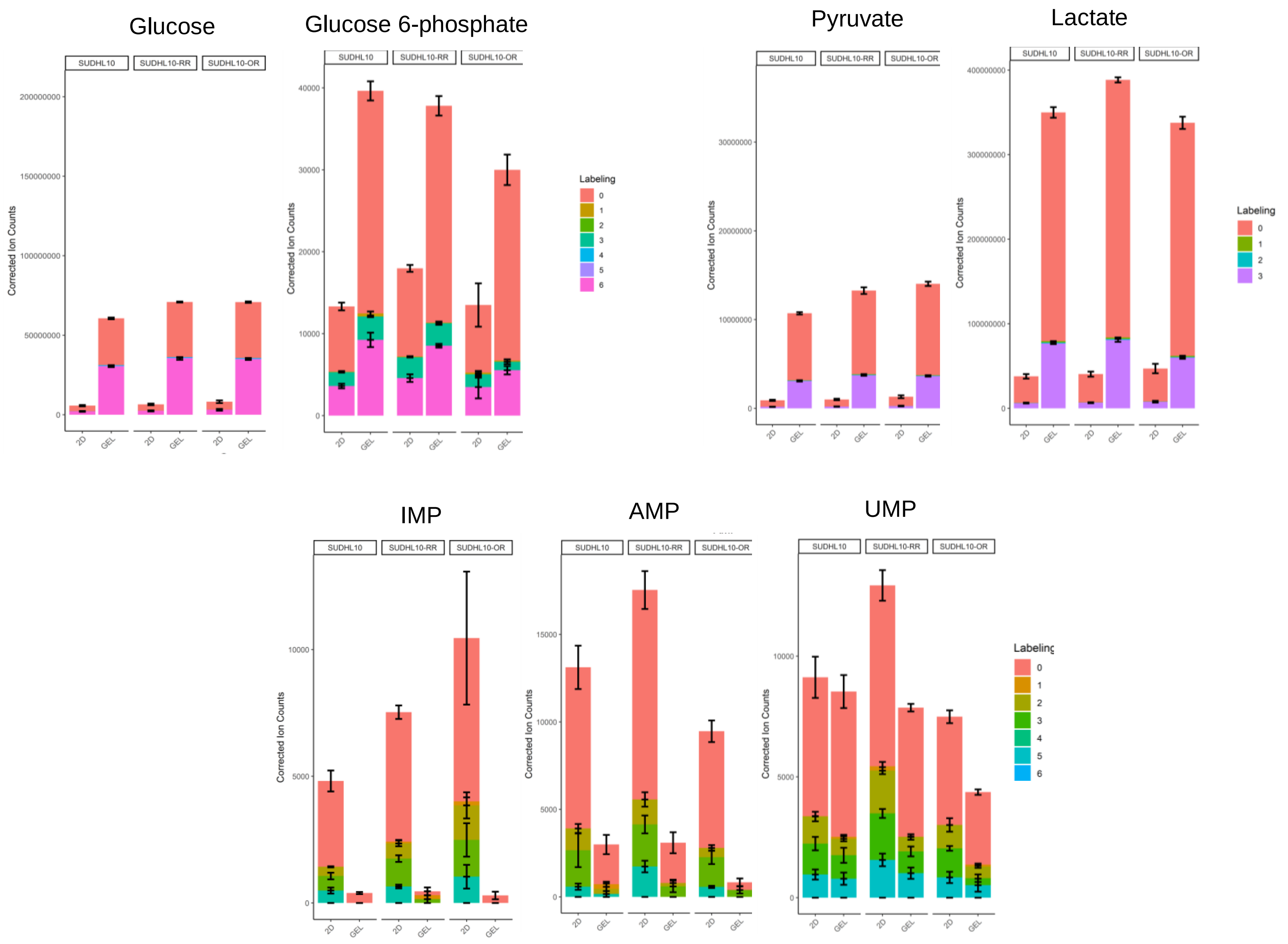

Supplementary Figure S3B: C^{13} Fractional Labeling

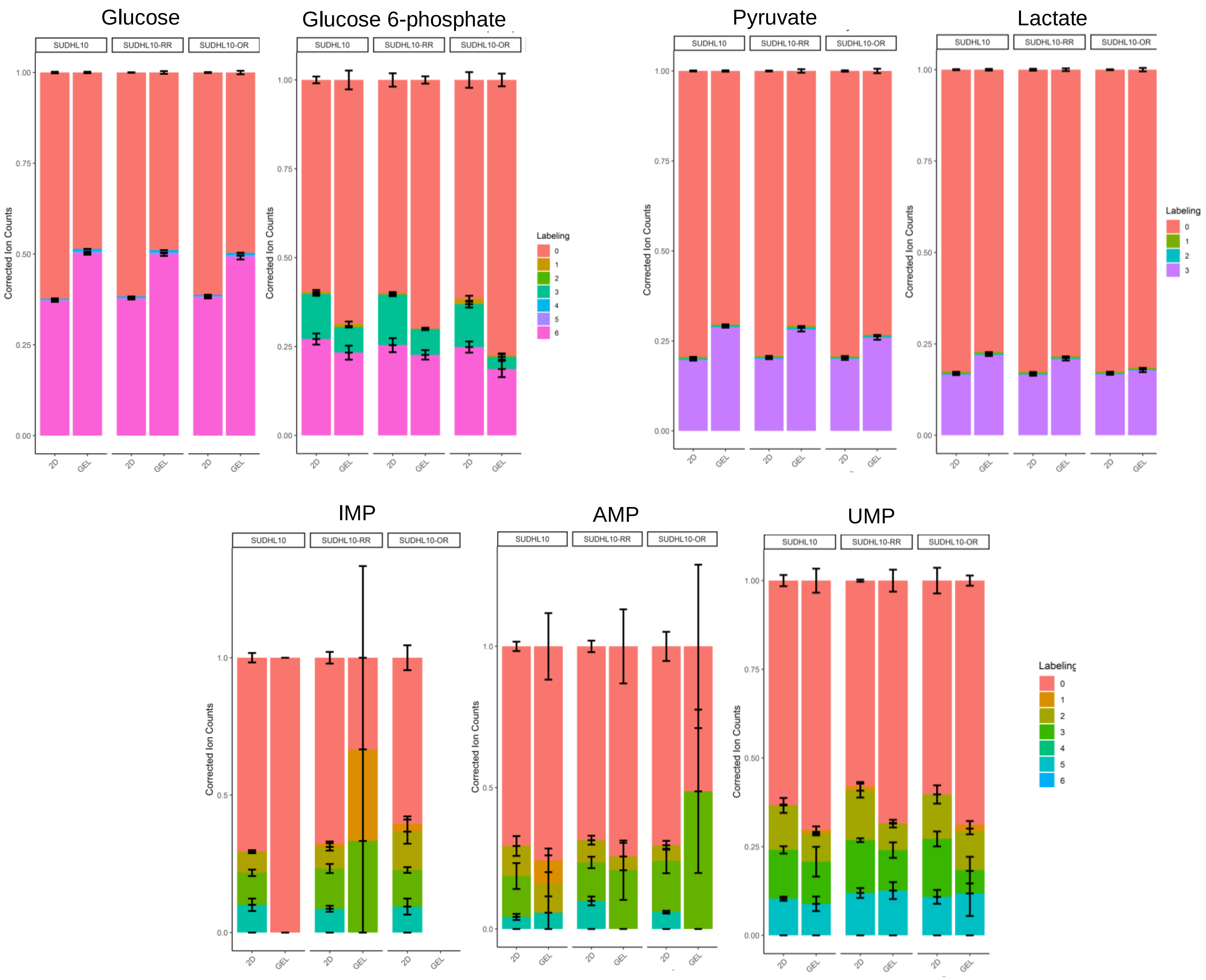

Supplement: Supplementary file 3 — Additional file 3: Figure S3. 13C isotope tracer labeling to elucidate metabolic flux variations between 2D and 3D grown DLBCL cell lines. (A) C13 labeled total pool (B) C13 fractional labelling. [file 40170_2022_291_MOESM3_ESM.pdf]
